# Determinants of postoperative acute kidney injury

**DOI:** 10.1186/cc7894

**Published:** 2009-05-22

**Authors:** Fernando José Abelha, Miguela Botelho, Vera Fernandes, Henrique Barros

**Affiliations:** 1 Department of Anesthesiology, Hospital de São João, Alameda Professor Hernani Monteiro, Porto, 4202-451, Portugal; 2 Department of Hygiene and Epidemiology, University of Porto Medical School, Alameda Professor Hernani Monteiro, Porto, 4202-451, Portugal

## Abstract

**Introduction:**

Development of acute kidney injury (AKI) during the perioperative period is associated with increases in morbidity and mortality. Our aim was to evaluate the incidence and determinants of postoperative AKI after major noncardiac surgery in patients with previously normal renal function.

**Methods:**

This retrospective cohort study was carried out in the multidisciplinary Post-Anaesthesia Care Unit (PACU) with five intensive care beds. The study population consisted of 1166 patients with no previous renal insufficiency who were admitted to these intensive care unit (ICU) beds over 2 years. After admission patients were followed for the development of AKI, defined as proposed by The Acute Kidney Injury Network (increment of serum creatinine [greater than or equal to] 0.3 mg/dL or 50% from baseline within 48 hours or urine output < 0.5 mL/kg/hr for > 6 hours despite fluid resuscitation when applicable). Patient preoperative characteristics, intraoperative management and outcome were evaluated for associations with acute kidney injury using an univariate and multiple logistic regression model.

**Results:**

A total of 1597 patients were admitted to the PACU and of these, 1166 met the inclusion criteria. Eighty-seven patients (7.5%) met AKI criteria. Univariate analysis identified age, American Society of Anesthesiologists (ASA) physical status, emergency surgery, high risk surgery, ischemic heart disease, congestive heart disease and Revised Cardiac Risk Index (RCRI) score as independent preoperative determinants for AKI in the postoperative period. Multivariate analysis identified ASA physical status, RCRI score, high risk surgery and congestive heart disease as preoperative determinants for AKI in the postoperative period. Patients that developed AKI had higher Simplified Acute Physiology Score (SAPS) II and Acute Physiology and Chronic Health Evaluation (APACHE) II, higher PACU length of stay (LOS), higher PACU mortality, higher hospital mortality and higher mortality at 6 months follow-up. AKI was an independent risk factor for hospital mortality (OR 3.12, 95% CI 1.41 to 6.93, *P *= 0.005).

**Conclusions:**

This study shows that age, emergency and high risk surgery, ischemic heart disease, congestive heart disease, ASA physical status and RCRI score were considered risk factors for the development of AKI, in patients needing intensive care after surgery. AKI has serious impact on PACU length of stay and mortality. AKI was an independent risk factor for hospital mortality.

## Introduction

Acute kidney injury (AKI) is commonly seen in the perioperative period and in the intensive care unit (ICU). It is associated with a prolonged hospital stay and high morbidity and mortality [1-6].

Acute renal failure is a complex disorder that occurs in a variety of settings with clinical manifestations ranging from a minimal elevation in serum creatinine to anuric renal failure.

To date, there is no universally accepted definition for acute kidney dysfunction. Varying terms, including *acute renal failure*, *renal insufficiency*, *kidney injury*, and *renal impairment*, and various definitions (e.g. percent or absolute increments of creatinine, or decrements of urine output) have been used in previous publications. Furthermore the term acute kidney injury has been put forth as the preferred nomenclature to replace acute renal failure with the understanding that the spectrum of AKI is broad and includes different degrees of severity.

We used the definition of AKI proposed by the Acute Kidney Injury Network (AKIN) that was formed to facilitate the development and execution of initiatives to ensure the best outcomes for patients with AKI [7]. As an initial step the AKIN group proposed the term acute kidney injury to reflect the entire spectrum of acute renal failure and developed interim diagnostic and staging criteria for AKI. The diagnostic criteria require a 0.3 mg/dL or 50% or higher change in serum creatinine (Scr) from baseline or a reduction on urine output of less than 0.5 ml/kg/hr over a six-hour interval, within a 48-hour period, following adequate volume resuscitation. These criteria were based on accumulating evidence that even small alterations in Scr are associated with severe consequences [2,4,8-10].

AKI occurs in approximately 1 to 5% of all hospitalized patients and is increasingly prevalent [1,11]. It is devastating to both patients and anesthesiologists, when patients with no evidence of renal dysfunction preoperatively develop AKI after surgery. Various studies have been published determining AKI incidence in specific patient populations: hospitalized patients [12], ICU patients [13,14], after cardiac surgery [15,16], patients with sepsis, and patients on renal replacement therapy [17,18].

Although predictors of postoperative acute renal failure after noncardiac surgery in patients with previously normal renal function had been studied previously [1], little is known about AKI predictors using the AKIN criteria.

The purpose of our study was to evaluate the incidence and determinants of the development of AKI in the immediate postoperative period in patients with previous normal renal function.

## Materials and methods

The Institutional Review Board of the Hospital de São João approved the study and waived the requirement for informed consent for the retrospective review of medical records. This retrospective cohort study was carried out at the Hospital São João, a 1124-bed community teaching hospital in Porto, Portugal, in the multidisciplinary post-anesthesia care unit (PACU). The PACU includes a surgical ICU with five beds in which surgical critically ill patients are admitted and closely monitored and treated.

All postoperative patients admitted to the surgical ICU area of the PACU, aged 18 years or more, who underwent scheduled or emergency noncardiac surgery between 1 March 2006 and 1 March 2008 with an overnight admission and more than 12 hours of PACU stay were eligible for the study. Patients were included only if a preoperative Scr within 30 days of the operative data was available. Patients readmitted during the study period were enrolled in relation to the time of their first admission. The PACU admits all surgical patients, with the exception of cardiothoracic patients.

Exclusion criteria were pre-existing renal dysfunction requiring renal replacement therapy or a preoperative Scr higher than 1.6 mg/dL for men and 1.4 mg/dL for women. Preoperative Scr values were defined as the most recent Scr (mg/dL) measured within 30 days of the surgery.

The primary outcome was development of AKI during PACU stay.

Patients were classified as having AKI if they had an increment of Scr of 0.3 mg/dL or higher or 50% or more increase within any 48-hour interval and/or an episode of less than 0.5 mL/kg/hr urine output for more than six hours despite fluid challenge of 500 mL or more normal saline, when appropriate.

The following variables were recorded on admission to the PACU: age, gender, body mass index (BMI), American Society of Anesthesiologists physical status (ASA-PS), preadmission comorbilities (specifically ischemic heart disease, congestive heart failure, cerebrovascular disease, hypertension, diabetes, hyperlipidemia), duration of anesthesia, type of anesthesia, core temperature, and troponin I blood levels.

Intraoperative data recorded for each case included administration of crystalloids, colloids, erythrocytes, and fresh frozen plasma.

Intraoperative and PACU data were collected as well as hospital length of stay (LOS). Mortality was recorded for all patients. The Acute Physiology and Chronic Health Evaluation (APACHE) II [19] and the Simplified Acute Physiology Score (SAPS) II [20] were calculated using standard methods.

Adapting a classification scheme developed by Lee and colleagues [21], we calculated the Revised Cardiac Risk Index (RCRI) score, assigning one point for each of the following risk factors: high-risk surgery (defined as intraperitoneal, intrathoracic, or suprainguinal vascular procedures), ischemic heart disease, congestive heart failure, cerebrovascular disease (defined as history of transient ischemic attack or history of cerebrovascular accident), and diabetes mellitus requiring insulin therapy.

Patients' demographics, and intraoperative and postoperative data were collected.

Physiologic data were recorded using customized data entry forms. Included was Scr that was recorded for each day and for PACU admission. These data were also recorded 24 hours before meeting criteria for AKI, at the time of AKI, 24 hours after AKI, and 48 hours or longer after AKI.

PACU and hospital LOS were also recorded. For mortality we have registered PACU mortality, hospital mortality, and mortality at six months after PACU discharge.

### Statistical analysis

Descriptive analyses of variables were used to summarize data and the Mann-Whitney U test was used to compare continuous variables; Chi-squared or Fisher's exact test were used to compare proportions between two groups of subjects.

To evaluate the determinants of postoperative AKI and to identify independent predictors of hospital mortality univariate analysis were performed using simple binary logistic regression with an odds ratio (OR) and 95% confidence interval (CI) with the following independent variables: age, gender, BMI, ASA-PS, type of surgery, comorbidities, RCRI score, type of anaesthesia, intraoperative fluid administration, troponin I blood levels at admission, length of anaesthesia, and temperature at admission (and AKI for mortality model). To reduce the risk of a type II error we have controlled the significance level for multiple comparisons applying the Bonferroni's correction for multiple comparisons (test-wise significance level divided by the number of tests performed). All variables were deemed to be significant if *P *≤ 0.002.

Multiple regression binary logistic with forward conditional elimination was used to examine covariate effects of each factor on AKI and to identify independent predictors of hospital mortality. In these models covariates with a univariate *P *≤ 0.002 in the respective univariate analysis were entered (applying the Bonferroni's correction for multiple comparisons).

Data were analyzed using SPSS for Windows version 16.0 (SPSS, Chicago, IL, USA).

## Results

A total of 1597 patients were admitted to the PACU during the study period and 1166 patients met the inclusion criteria and were followed for the development of AKI after PACU admission. One-hundred and twenty-one patients were excluded because they had abnormal renal function preoperatively (defined as Scr higher than 1.6 mg/dL for men and 1.4 mg/dL for women); 196 were excluded because they stayed less than 12 hours and did not had an overnight admission in the PACU; 52 were excluded because they were less than 18 years of age; 44 were admitted more than once to the PACU and therefore excluded; 12 were excluded because they were not surgical patients; and 6 had no preoperative Scr measurement and were excluded.

The remaining 1166 were followed for the development of AKI after PACU admission. Eighty-seven (7.5%) developed AKI. The characteristics of patients with and without AKI are summarized in Table 1. Patients with AKI were older (median age 68 versus 64 years, *P *= 0.002), had lower BMI (median 24 versus 25, *P *= 0.020), were more likely to have been submitted for general anesthesia (85% versus 80%, *P *= 0.004), had higher troponin I at admission (0.18 ± 0.52 versus 0.06 ± 0.29, *P *= 0.011), were more likely to have been submitted to emergency surgery (33% versus 19%, *P *= 0.001) or high risk surgery (72% versus 43%, *P *< 0.001), had more frequently ischemic heart disease (38% versus 23%, *P *= 0.001) and congestive heart disease (46% versus 18%, *P *< 0.001), were more likely to be ASA-PS IV/V (22% versus 5%, *P *< 0.001)and have RCRI scores of more than 2 (74% versus 62%, *P *< 0.001), and had higher volume of intraoperative fluids administered (3.0 ± 2.3 versus 2.6 ± 1.7, *P *= 0.021 for crystalloids; 0.3 ± 0.5 versus 0.2 ± 0.4, *P *= 0.014 for colloids; 1.2 ± 2.6 versus 0.7 ± 1.5, *P *= 0.003 for units of erythrocytes; 0.6 ± 1.5 versus 0.2 ± 1.0, *P *= 0.004 for units of fresh frozen plasma).

**Table 1:** Patient characteristics and outcomes

**Variable**	**All patients** **(n = 1166)**	**AKI** **(n = 87)**	**Non AKI** **n = 1079**	***P***
Age in years, median (IQR)	64 (53 to 73)	68 (57 to 76)	64 (53 to 73)	0.002
Age group, n (%)				0.003
≥65 years	581 (50)	56 (64)	525 (49)	
< 65 years	585 (50)	31 (36)	554 (51)	
Sex, n (%)				0.470
Male	761 (65)	56 (64)	705 (65)	
Female	405 (35)	31 (36)	374 (35)	
ASA physical status				< 0.001
I/II/III	1094 (94)	68 (78)	1026 (95)	
IV/V	72 (6)	19 (22)	53 (5)	
Body mass index in Kg/m^2^, median (IQR)	25 (22 to 28)	24 (22 to 27)	25 (22 to 28)	0.020
General anesthesia, n. (%)	940 (81)	72 (85)	868 (80)	0.004
Duration of anesthesia (min.) median (IQR)	210 (150 to 300)	200 (125 to 300)	210 (150 to 300)	0.772
Temperature at admission on PACU, mean ± sd	35.23 ± 1.31	35.04 ± 1.70	35.25 ± 1.27	0.251
Troponin I at admission, mean ± sd	0.07 ± 0.32	0.18 ± 0.52	0.06 ± 0.29	0.011
Hypertension, n (%)	565 (49)	40 (46)	525 (49)	0.356
Hyperlipidemia, n (%)	338 (29)	24 (28)	314 (29)	0.438
Emergency surgery	231 (20)	29 (33)	202 (19)	0.001
High-risk surgery, n (%)	528 (45)	63 (72)	465 (43)	< 0.001
Ischemic heart disease, n (%)	279 (24)	33 (38)	246 (23)	0.001
Congestive heart disease, n (%)	233 (20)	40 (46)	193 (18)	< 0.001
Cerebrovascular disease, n (%)	170 (15)	12 (11)	158 (15)	0.490
Insulin therapy for diabetes, n (%)	104 (9)	9 (10)	95 (9)	0.370
Total RCRI				< 0.001
≤2	437 (38)	54 (62)	833 (77)	
> 2	729 (63)	33 (28)	246 (23)	
Intraoperative fluid volume				
Chrystaloids (L) (IQR)	2.6 ± 1.8 (1.2 to 3.5)	3.0 ± 2.3 (1.5 to 3.6)	2.6 ± 1.7 (1.2 to 3.4)	0.021
Colloids (L) (IQR)	0.2 ± 0.4 (0 to 0.5)	0.3 ± 0.5 (0 to 0.5)	0.2 ± 0.4 (0 to 0.5)	0.014
Erythrocytes (Units) (IQR)	0.7 ± 1.6 (0 to 1)	1.2 ± 2.6 (0 to 2)	0.7 ± 1.5 (0 to 1)	0.003
Fresh frozen plasma (Units) (IQR)	0.3 ± 1.0 (0 to 0)	0.6 ± 1.5 (0 to 0)	0.2 ± 1.0 (0 to 0)	0.004

AKI = acute kidney injury; ASA = American Society of anesthesiologists, IQR = interquartile range; PACU = post anesthesia care unit; RCRI = Revised Cardiac Risk Index; SD = standard deviation.

Table 2 shows the severity of disease scores and outcome for patients with and without AKI. Patients with AKI were more severely ill (median SAPS II 29 versus 18, *P *< 0.001 and median APACHE II 12 versus 7, *P *< 0.001), and stayed longer in the PACU (median LOS 38 versus 25, *P *< 0.001). For the six months follow-up we obtained the postoperative vital status of all patients. The unadjusted mortality rate at six months follow-up of patients with AKI was 36%, nearly four times the mortality rate of those without AKI (36% versus 10%, *P *< 0.001). The increased mortality observed among patients with AKI was even greater for hospital mortality (26%, versus 3%, *P *< 0.001), and PACU mortality (17% versus 1%, *P *< 0.001).

**Table 2:** Severity of disease scores, PACU and hospital length of stay, and mortality

**Variable**	**All patients** **(n = 1166)**	**AKI** **(n = 87)**	**Non AKI** **(n = 1079)**	***P***
SAPS II, median (IQR)	18 (12 to 25)	29 (20 to 45)	18 (12 to 24)	< 0.001
APACHE II, median (IQR)	8 (5 to 11)	12 (8 to 17)	7 (5 to 10)	< 0.001
PACU length of stay (hours), median (IQR)	25 (21 to 46)	38 (22 to 72)	25 (21 to 44)	< 0.001
Hospital length of stay (days), median (IQR)	13 (7 to 28)	16 (8 to 32)	13 (7 to 28)	0.793
Mortality in PACU, n (%)	23 (2.0)	17 (17.2)	8 (0.7)	< 0.001
Mortality in hospital, n (%)	50 (4.3)	23 (26.4)	27 (2.5)	< 0.001
Mortality at six months follow-up, n (%)	134 (11.5)	31 (35.6)	103 (9.5)	< 0.001

AKI = acute kidney injury; APACHE II = Acute Physiology and Chronic Health Evaluation; IQR = interquartile range; PACU = post anesthesia care unit; SAPS II = Simplified Acute Physiology Score.

Univariate analysis for determinants of AKI and their relevant *P*-values are summarized in Table 3. Univariate analysis identified the following independent predictors for development of AKI in the immediate postoperative period (*P *≤ 0.002): age (OR 1.03, 95% CI 1.01 to 1.04, *P *= 0.002), emergency surgery (OR 2.17, 95% CI 1.36 to 3.49, *P *= 0.001), ASA-PS (OR 5.41, 95% CI 3.03 to 9.65, *P *< 0.001 for ASA-PS IV/V patients), high-risk surgery (OR 3.47, 95% CI 2.13 to 5.63, *P *< 0.001), ischemic heart disease (OR 2.07, 95% CI 1.31 to 3.26, *P *= 0.002), congestive heart disease (OR 3.91, 95% CI 2.49 to 6.12, *P *< 0.001), and RCRI score (OR 3.30, 95% CI 2.12 to 5.14, *P *< 0.001 for RCRI > 2).

**Table 3:** Univariate analysis for determinants of renal dysfunction

**Variable**	**AKI/non AKI**	**Odds ratio (95% CI)**	***P***
			
	**n = 87/n = 1079**		
Age	66.9 ± 13.2/61.5 ± 15.5	1.03 (1.01 to 1.04)	0.002
≥65 years	56 (64)/525 (49)	1.91 (1.21 to 3.00)	0.005
< 65 years	31 (36)/554(51)	1	
Gender		0.96 (0.61 to 1.51)	0.855
Female	31 (36)/374 (35)		
Male	56 (64)/705 (65)		
Body mass index, median	24.0 ± 4.3/25.6 ± 5.8	0.94 (0.89 to 1.00)	0.019
Duration of anesthesia (min.)	233 ± 140/230 ± 113	1.00 (0.99 to 1.00)	0.772
General/Combined anesthesia, n. (%)	72 (85)/868 (80)	4.59 (1.43 to 14.71)	0.010
Emergency surgery	29 (33)/202 (19)	2.17 (1.36 to 3.49)	0.001
ASA physical status			
II/III	68(78)/1026 (95)	1	
IV/V	19 (22)/53 (5)	5.41 (3.03 to 9.65)	< 0.001
Temperature at admission	35.1 ± 1.8/35.2 ± 1.3	0.91 (0.78 to 1.07)	0.251
Troponin at admission	0.20 ± 0.57/0.06 ± 0.29	1.81 (0.95 to 3.44)	0.070
Hypertension	40(46)/525 (49)	0.90 (0.58 to 1.39)	0.631
Hyperlipidemia	24 (28)/314(29)	0.93 (0.57 to 1.50)	0.765
High-risk surgery	63 (72)/465 (43)	3.47 (2.13 to 5.63)	< 0.001
Ischemic heart disease	33 (38)/246 (23)	2.07 (1.31 to 3.26)	0.002
Congestive heart disease	40 (46)/193 (18)	3.91 (2.49 to 6.12)	< 0.001
Cerebrovascular disease	12(14)/158 (15)	0.93 (0.50 to 1.76)	0.829
Insulin therapy for diabetes	9 (10)/95 (9)	1.20 (0.58 to 2.46)	0.628
RCRI			
≤2	54 (62)/833 (77)	1	
> 2	33 (38)/246 (23)	3.30 (2.12 to 5.14)	< 0.001
Intraoperative fluid volume			
Crystalloids (L)	3.0 ± 2.3/2.6 ± 1.7	1.14 (1.02 to 1.28)	0.024
Colloids (L)	0.3 ± 0.5/0.2 ± 0.4	1.76 (1.11 to 2.78)	0.016
Erythrocytes (Units)	1.2 ± 2.6/0.7 ± 1.5	1.15 (1.04 to 1.27)	0.005
Fresh frozen plasma (Units)	0.6 ± 1.5/0.2 ± 1.0	1.22 (1.04 to 1.42)	0.012

AKI = acute kidney injury; ASA = American Society of Anesthesiologists; CI = confidence interval; RCRI = Revised Cardiac Risk Index

Univariate analysis for severity of disease, LOS, and mortality are summarized in table 4. Severity of disease scores SAPS II and APACHE II were significantly higher in AKI patients (OR 1.08, 95% CI 1.06 to 1.10, *P *< 0.001 and OR 1.18, 95% CI 1.14 to 1.23, *P *< 0.001 respectively). The perioperative onset of AKI in patients with previously normal renal function was associated with significantly increased in PACU LOS (OR 1.14, 95% CI 1.06 to 1.21, *P *< 0.001), higher PACU mortality (OR 27.89, 95% CI 11.45 to 67.96, *P *< 0.001), higher hospital mortality (OR 14.00, 95% CI 7.60 to 25.79, *P *< 0.001) and higher mortality at six months follow-up (OR 5.25, 95% CI 3.24 to 8.51, *P *< 0.001).

**Table 4:** Univariate analysis for severity of disease, length of stay, and mortality

**Variable**	**AKI/non AKI**	**Odds ratio (95% CI)**	***P***
			
	**n = 87/n = 1079**		
SAPS II	33.3 ± 18.7/18.9 ± 10.3	1.08 (1.06 to 1.10)	< 0.001
APACHE II	13.0 ± 6.8/8.0 ± 4.3	1.18 (1.14 to 1.23)	< 0.001
PACU stay (days)	2.8 ± 3.4/1.8 ± 2.1	1.14 (1.06 to 1.21)	< 0.001
Hospital stay (days)	25 ± 27/24 ± 32	1.00 (0.99 to 1.01)	0.793
Mortality in the PACU	17 (17)/8 (1)	27.89 (11.45 to 67.96)	< 0.001
Mortality in the hospital	23 (26)/27 (3)	14.00 (7.60 to 25.79)	< 0.001
Mortality at 6 months	31 (36)/103 (10)	5.25 (3.24 to 8.51)	< 0.001

AKI = acute kidney injury; APACHE II = Acute Physiology and Chronic Health Evaluation; CI = confidence interval; PACU = post anesthesia care unit; SAPS = Simplified Acute Physiology Score.

Multiple regression logistic analysis was used to examine covariate effects of each factor on AKI (Table 5). This regression model included all variables with statistical significance in the univariate analysis made for determinants of AKI development. This analysis showed that significant risk factors for AKI were ASA-PS (OR 3.94, 95% CI 2.07 to 7.51, *P *< 0.001, for ASA-PS IV/V patients), RCRI (OR 2.45, 95% CI 1.52 to 3.96, *P *< 0.001 for RCRI > 2), high-risk surgery (OR 3.34, 95% CI 2.02 to 5.33, *P *< 0.001), and congestive heart disease (OR 2.34, 95% CI 1.42 to 3.88, *P *= 0.001).

**Table 5:** Multivariate analysis to evaluate covariate effects on AKI development

**Variable**	**Beta**	**Adjusted OR**	**p**
ASA physical status	1.371	3.94 (2.07 to 7.51)	< 0.001
RCRI	0.896	2.45 (1.52 to 3.96)	< 0.001
High-risk-surgery	1.206	3.34 (2.02 to 5.53)	< 0.001
Congestive Heart disease	0.851	2.34 (1.42 to 3.88)	0.001

AKI = acute kidney injury; ASA = American Society of Anesthesiologists; RCRI = Revised Cardiac Risk Index.

Multiple logistic regression analyses was used to examine covariate effects of each factor on hospital mortality (Table 6). The regression model included all variables with statistical significance in the univariate analysis for determinants of hospital mortality. This analysis showed that AKI was an independent risk factors for hospital mortality (OR 3.12, 95% CI 1.41 to 6.93, *P *= 0.005) after adjustment for age, ASA-PS, high-risk surgery, congestive heart failure, emergency surgery, SAPS II, APACHE II, PACU LOS, RCRI, and AKI. Other independent predictors of hospital mortality were ASA-PS (OR 5.17, 95% CI 2.38 to 11.21, *P *< 0.001, for ASA-PS IV/V patients), high-risk surgery (OR 2.15, 95% CI 1.02 to 4.53, *P *= 0.043), congestive heart disease (OR 2.90, 95% CI 1.45 to 5.76, *P *= 0.002), and SAPS II (OR 1.06, 95% CI 1.03 to 1.08, *P *< 0.001).

**Table 6:** Multivariate regression analysis for predictors of in-hospital mortality

Variable	**Simple OR**	***P***	Adjusted* OR (95% CI)	***P* ^a^**
Age group, n (%)				
≥65 years	2.69	0.002		
< 65 years	1			
ASA physical status				
I/II/III	1		1	
IV/V	15.12	< 0.001	5.17 (2.38 to 11.21)	< 0.001
Temperature	0.71	< 0.001		
Emergency surgery	4.05	< 0.001		
High-risk surgery	3.26	< 0.001	2.15 (1.02 to 4.53)	0.043
Congestive heart disease	6.75	< 0.001	2.90 (1.45 to 5.76)	0.002
SAPS II	1.09	< 0.001	1.06 (1.03 to 1.08)	< 0.001
APACHE II	1.27	< 0.001		
PACU LOS (days)	1.19	< 0.001		
AKI	14.00	< 0.001	3.12 (1.41 to 6.93)	0.005

^a^Logistic regression analysis with stepwise forward method was used with an entry criterion of *P *< 0.05 and a removal criterion of *P *> 0.1.

*Adjusted to age, ASA physical status, high-risk surgery, congestive heart failure, emergency surgery, SAPS II, APACHE II, PACU LOS, and AKI.

AKI = acute kidney injury; APACHE II = Acute Physiology and Chronic Health Evaluation; ASA = American Society of Anesthesiologists; CI = confidence interval; LOS = length of stay; OR = odds ratio; PACU = post anesthesia care unit; SAPS = Simplified Acute Physiology Score.

## Discussion

We report the incidence of postoperative AKI among patients with normal preoperative renal function to be 7.5%. This incidence is consistent with that reported among general hospitalized patients, ranging from 1 to 5% [1]; although, in other studies, the incidence of postoperative acute renal failure varies from 1.1 to 17% [1] depending on the definition of acute renal failure [22]. Acute renal failure remains a medical problem with a daunting outcome: even the best centres typically report mortalities of 50 to 80% [23]. Recent epidemiologic studies demonstrate the wide variation in etiologies and risk factors [24,25].

Unfortunately, acute renal failure is often unrecognized as definitions for the disease range from quantitative and qualitative alterations in Scr to alterations in urine output and dialysis requirement. The lack of a universally recognized definition of ARF has posed a significant limitation contributing to the lack of clinical success. Recognizing the need for uniform standards, the Acute Dialysis Quality Initiative group in 2002 proposed consensus recommendations of the Risk, Injury, Failure, Loss and End-stage kidney disease (RIFLE) criteria for the definition and staging of acute renal failure [10]. The wide interest sparked by the publication of these criteria demonstrated the additional need for multidisciplinary collaborative efforts for clinical and translational research in this area. Consequently, the AKIN was formed to facilitate the development and execution of initiatives to ensure the best outcomes for patients with AKI. As an initial step the AKIN group proposed the term acute kidney injury to reflect the entire spectrum of acute renal failure and developed interim diagnostic and staging criteria for AKI. The diagnostic criteria require a 0.3 mg/dL or a 50% or higher change in Scr from baseline or a reduction in urine output of less than 0.5 ml/kg/hr over a six-hour interval, within a 48-hour period, following adequate volume resuscitation. These criteria were based on accumulating evidence that even small alterations in Scr are associated with severe consequences [2,10,26] and the RIFLE stage correlated with adverse events [27]. Validation of these concepts was now provided by the study of Barrantes and colleagues [13] in a consecutive cohort of patients admitted to a general medical ICU. The overall incidence of AKI in this cohort is much higher (25.4 to 44.6%) than described in our current study.

Postoperative AKI remains a leading cause of morbidity, mortality, prolonged hospital stay, and increased hospital cost. In this study, we have attempted to correlate and predict factors (preoperative, intraoperative, and early postoperative) predisposing to AKI. By doing so, one might be able to predict those at high risk and interventional measures might be planned in advance to improve outcome after such a complication, achieving a better outcome and a more efficient use of hospital and intensive care resources.

We intentionally excluded patients with documented preoperatively raised Scr in order to predict AKI among patients with preoperatively normal renal function, as dictated by a Scr less than 1.6 mg/dL for men and 1.4 mg/dL for women.

There have been a variety of predictive models developed to stratify risk in patients undergoing cardiac surgery [3,28] and there is an important recently published study addressing renal dysfunction after noncardiac surgery [1]. These studies identified the incidence and risk factors for postoperative acute renal failure after surgery using acute renal failure definitions that are more strict and less sensitive for renal insufficiency than those used in our study for AKI. Most predictors or determinants we have found in our study are consistent with those described in these studies: emergent surgery, advanced age, peripheral vascular occlusive disease, and high-risk surgery.

In his study with similar objectives but with different methodology Kheterpal and colleagues [1] found seven independent preoperative predictors for the development of acute renal failure after noncardiac surgery in patients with previously normal renal function and four of these predictors were similarly found in our study: age, emergency surgery, high-risk surgery, and ischemic heart disease. Beyond these we also found congestive heart disease, ASA-PS, and higher RCRI scores as risk factors for the development of AKI.

The ASA-PS score, a preoperative evaluation used routinely for every patient, was never intended to be a perioperative risk score, but all large-scale studies have suggested that a high ASA-PS score is one of the best predictors of postoperative morbidity [29,30]. This indicator itself depends on a more generic classification of the presence of disease, and in itself indicates the presence of comorbidities. ASA-PS classification is a strong prognostic predictor for the development of postoperative medical complications for patients in a perioperative setting and in fact in our study, we found that increasingly severe systemic disease (higher ASA-PS level) is a determinant of AKI.

The RCRI is a prediction tool for major cardiac complications after noncardiac surgery [21,31]. It was developed for the prediction of cardiac risk based on six independent prognostic factors: high-risk surgery, ischemic heart disease, congestive heart disease, history of cerebrovascular disease, insulin therapy for diabetes, and preoperative Scr higher than 2.0 mg/dL. RCRI score is based on predictors, which independently have a significant association with cardiovascular events. This is probably the reason why RCRI score is a predictor of AKI in immediate postoperative period and the meaning of that could be that patients with pre-existing cardiovascular disease have an increased perioperative risk of developing AKI in postoperative period. In the present study, we found that RCRI score was an independent predictor for development of AKI as well as some of the factors included in it, showing that they were independent predictors for AKI.

In fact, to assume that both cardiac and renal complications are based on ischemic injury to sensitive organs may be a plausible explanation for RCRI as an index. Even some of the individualized risk factors that compose it appears as a risk factor for postoperative renal failure.

We noted congestive heart failure to be an independent predictor for hospital-acquired AKI as stated by Drawz and colleagues [32] and that in an ICU population of the study of Barrantes and colleagues [13] patients with congestive heart failure were more likely to develop AKI.

Acute renal injury without the need for renal replacement therapy is associated with increased mortality in critically ill patients and in postoperative cardiac and noncardiac surgery [1,13,33,34]. Similarly our data suggest a relation between AKI and increased postoperative mortality after noncardiac surgery; this increased mortality rate was observed in the PACU, in the hospital, and at six months after PACU discharge.

Mortality of patients with acute renal failure is high. In the study by Barrantes and colleagues [13], the authors demonstrated that critical care patients meeting the definition for AKI similar to the definition used in our study, had a hospital mortality of 46% and that these patients were three times more likely to die during hospitalization. Our hospital mortality were lower (27%) but AKI patients were 13 times more likely to die during hospitalization and 26 times more likely to die during PACU stay.

In our study patients with AKI had significantly higher hospital mortality than patients without AKI. Even when controlled for other variables with a multiple variable regression analysis, AKI remains an independent risk factor for hospital mortality. This is consistent with previous data [1,6,35].

The study by Chertow and colleagues [2] first demonstrated that a change of Scr 0.3 mg/dL or more at any time during hospitalization was associated with increased mortality. Our study, using the same definition for AKI used in the study by Barrantes and colleagues [13], not only supports that such small changes in creatinine are associated with meaningful differences in outcome but also that acute increments of 0.3 mg/dL in a 48-hour period predict mortality.

As in the previous focused study by Barrantes and colleagues [13], patients with AKI had a longer duration of PACU stay.

We should note some limitations to our analysis. First, our study was retrospective in nature; we were unable to identify and analyse all potential confounding factors. The data were collected as part of the delivered clinical cares. As a result, the data reflect the electronic medical record, and no additional detail is available. Documentation of urine output, the use of vasoactive substances and hemodynamic stability during surgery was less reliable. In our analysis we could only quantify volume of intraoperative fluid administration and we were unable to address the complete role of intravenous hydration in AKI because several data elements involved in the quantification of resuscitation volume were not accurately collected in the medical records. This might be an important aspect of pathophysiology for AKI development and could not be included in our analysis.

In the study we do not analyse time points for AKI development and we did not make exclusion criteria to prevent the admission of patients with late development of AKI. With this approach we could have considered AKI that could have been due to complications other than surgery. We did not assess other potential consequential effects of AKI and the studied outcomes (mortality and LOS) may not necessarily capture all relevant consequences of AKI. The mortality data are based on all-cause mortality and no additional detail regarding the cause of death are available for review. This may limit the ability to interpret our data on mortality.

## Conclusions

Using the interim consensus definition of AKI we could predict meaningful clinical outcomes and were able to identify risk factors for the development of AKI in patients needing intensive care after surgery.

## Key messages

• AKI is commonly seen in the postoperative period after major surgery. In our study 7.5% of patients developed AKI after surgery.

• Age, emergency surgery, ASA-PS, high-risk surgery, ischemic heart disease, congestive heart disease, and total RCRI score were considered independent predictors for the development of AKI.

• Patients that developed AKI stayed longer in the PACU.

• The perioperative onset of AKI was associated with significantly increased PACU LOS and higher mortality rates at the hospital and at six months follow-up.

• AKI was an independent risk factor for hospital mortality.

## Abbreviations

AKI: acute kidney injury; AKIN: Acute Kidney Injury Network; APACHE II: Acute Physiology and Chronic Health Evaluation; ASA-PS: American Society of Anesthesiologists physical status; BMI: body mass index; CI: confidence interval; ICU: intensive care unit; LOS: length of stay; OR: odds ratio; PACU: post anesthesia care unit; RCRI: Revised Cardiac Risk Index; RIFLE: Risk, Injury, Failure, Loss and End-stage kidney disease; SAPS: Simplified Acute Physiology Score; Scr: serum creatinine.

## Competing interests

The authors declare that they have no competing interests.

## Authors' contributions

FA participated in conception, design, acquisition of the data, analysis of the data, statistical analysis, critical revision of the manuscript, and supervision. MB and VF participated in conception, design, acquisition of the data, analysis of the data, and critical revision of the manuscript. HB was involved in drafting the manuscript, analysis of the data, and revising it critically for important content. All authors read and approved the final manuscript.
